# Structural assessment of SARS-CoV2 accessory protein ORF7a predicts LFA-1 and Mac-1 binding potential

**DOI:** 10.1042/BSR20203837

**Published:** 2021-01-08

**Authors:** Zubair Ahmed Nizamudeen, Emma-Ruoqi Xu, Vivin Karthik, Mohamed Halawa, Kenton P. Arkill, Andrew M. Jackson, David O. Bates, Jonas Emsley

**Affiliations:** 1 Division of Cancer and Stem Cells, School of Medicine, Biodiscovery Institute, University of Nottingham, Nottingham NG7 2RD, U.K.; 2 School of Pharmacy, Biodiscovery Institute, University of Nottingham, Nottingham NG7 2RD, U.K.; 3 Graduate School of Biomedical Sciences and Engineering, University of Maine, Orono, ME 04469, U.S.A.

**Keywords:** COVID19, LFA1, Mac1, SARS-Cov2

## Abstract

ORF7a is an accessory protein common to SARS-CoV1 and the recently discovered SARS-CoV2, which is causing the COVID-19 pandemic. The ORF7a protein has a structural homology with ICAM-1 which binds to the T lymphocyte integrin receptor LFA-1. As COVID-19 has a strong immune component as part of the disease, we sought to determine whether SARS-CoV2 would have a similar structural interaction with LFA-1. Using molecular docking simulations, we found that SARS-CoV2 ORF7a has the key structural determinants required to bind LFA-1 but also the related leukocyte integrin Mac-1, which is also known to be expressed by macrophages. Our study shows that SARS-CoV2 ORF7a protein has a conserved Ig immunoglobulin-like fold containing an integrin binding site that provides a mechanistic hypothesis for SARS-CoV2’s interaction with the human immune system. This suggests that experimental investigation of ORF7a-mediated effects on immune cells such as T lymphocytes and macrophages (leukocytes) could help understand the disease further and develop effective treatments.

## Introduction

Severe acute respiratory syndrome coronavirus 2 (SARS-CoV2) is a newly discovered pathogen responsible for the COVID-19 pandemic (2019–2020) with a total of 77,471,325 cases worldwide resulting in 1,705,008 deaths as of December 22, 2020 (https://coronavirus.jhu.edu/map.html). SARS-CoV2 shares high similarity with the previously identified SARS-CoV1. The viral genome of both SARS-CoV1 and SARS-CoV2 is consisted of a positive-strand RNA that codes for characteristic proteins: replicase, spike, membrane, envelope, nucleocapsid and accessory gene encoded proteins. Pairwise alignment shows RNA genome sequence similarity of 78.8% between SARS-CoV1 (NC_004718.3) and SARS-CoV2 (NC_045512.2). Moreover, multiple studies have confirmed that both SARS-CoV1 and SARS-CoV2 use angiotensin 2 (ACE2) receptor as a common cellular entry pathway in humans [1–3]. Therefore, the genomic and proteomic data generated over the last decade since SARS-CoV1 outbreak can be useful to test and develop treatment strategies for the current pandemic caused by SARS-CoV2.

In the present study, we have focused on specific structural properties of the open reading frame 7a (ORF7a) accessory protein of SARS-CoV2, based on previously published data on SARS-CoV1 [4,5]. Sequence analysis predictions reported ORF7a as a type I transmembrane protein of 122 amino acid in length (15 residue N-terminal signal peptide, 81 residue luminal domain, 21 residue transmembrane segment, and a 5 residue cytoplasmic tail), and is unique to SARS-CoV [5]. Like most other accessory proteins of SARS-CoV genome, ORF7a is dispensable for viral replication *in vitro* [6–9], but its deletion has been suggested to lead to viral attenuation *in vivo* [5,10]. SARS-CoV1-ORF7a protein constitutes an immunoglobulin-like fold and has topological similarity to domain 1 of human intercellular adhesion molecule-1 (ICAM-1) [4,5]. ICAM-1 is known to bind and interact with leukocyte markers (lymphocyte function-associated antigen or LFA-1 or αLβ2) and Macrophage-1 antigen (integrin αMβ2 or macrophage integrin or Mac-1) [11–14]. For the majority of LFA-1 and Mac-1 interactions described, the α-subunit inserted domain, or I-domain, is the principal ligand-binding domain and contains an unusual Mg^2+^ binding site on its surface at the top of the β-sheet termed the metal ion-dependent adhesion site (MIDAS), which is capable of accepting a donor acidic amino acid sidechain from co-ordinating ligands [4]. Like ICAM-1, ORF7a protein can bind to LFA-1 [15] which plays a critical role in leukocyte function and regulates T-cell activation [16–20]. This suggested early on that SARS-CoV1 can interact with leukocytes via ORF7a protein and thereby can play a direct role in modulating the immune system and inflammation. Here we compare the sequence, structure and molecular docking characteristics of ORF7a in SARS-CoV1 and SARS-CoV2, with leukocyte markers LFA-1 and Mac-1.

## Materials and methods

Three-dimensional models of the ORF7a:integrin docked complex structures were calculated using the High Ambiguity Driven protein–protein Docking HADDOCK 2.2 docking program [21] with the available ORF7a crystal structure (PDB code: 6W37) reported at 2.9 Å resolution. Multiple crystal structures are available for the integrin LFA-1 I-domain in the open conformation and PDB code: 1T0P was selected which at 1.7 Å resolution was the highest resolution structure available. Highly similar results were observed for other LFA-1 I-domain structures when entered into docking with the ORF7a crystal structure.

Information was introduced as ambiguous interaction restraints (AIRs) to drive the docking process in the form of residues from the MIDAS face of the integrin I domain. For LFA-1 these residues included the MIDAS metal ion and for ORF7a residue E26. Active residues were defined based on the integrin I-domain MIDAS metal ion and residue E26 from ORF7a. Passive residues surrounding the active residues were assigned automatically by HADDOCK. For the LFA-1 I-domain docking to ORF7a the highest ranked score was −72.0 with a cluster size of 130 and this solution is shown in Figure 2A. The second highest ranking had a score of −57.4 and a reduced cluster size of 15. As a frame of reference for these values docking of the crystal structure for the native cognate ligand ICAM-3 onto the LFA-1 I domain resulted in the highest ranked score of -98.6 with a cluster size of 163. The second highest ranking had a score of −43.9 and a reduced cluster size of 12. Multiple templates were also available for the integrin for the Mac-1 I-domain in the open conformation and PDB code: 1ID0 was selected which at 1.7 Å resolution was the highest resolution structure available. The Mac-1 I-domain docking to ORF7a was conducted in a similar fashion and AIRs were defined using active residues were defined based on the integrin I-domain MIDAS metal ion and residue E26 from ORF7a. Passive residues surrounding the active residues were assigned automatically by HADDOCK. The highest ranked score was -78.4 with a cluster size of 131 and this Mac-1/ORF7a complex is shown in Figure 2B. The second highest ranking had a score of -55.3 and a cluster size of 14.

## Results

### Sequence and structural alignment of SARS-CoV1-ORF7a and SARS-CoV2-ORF7a proteins

**Figure 1:**
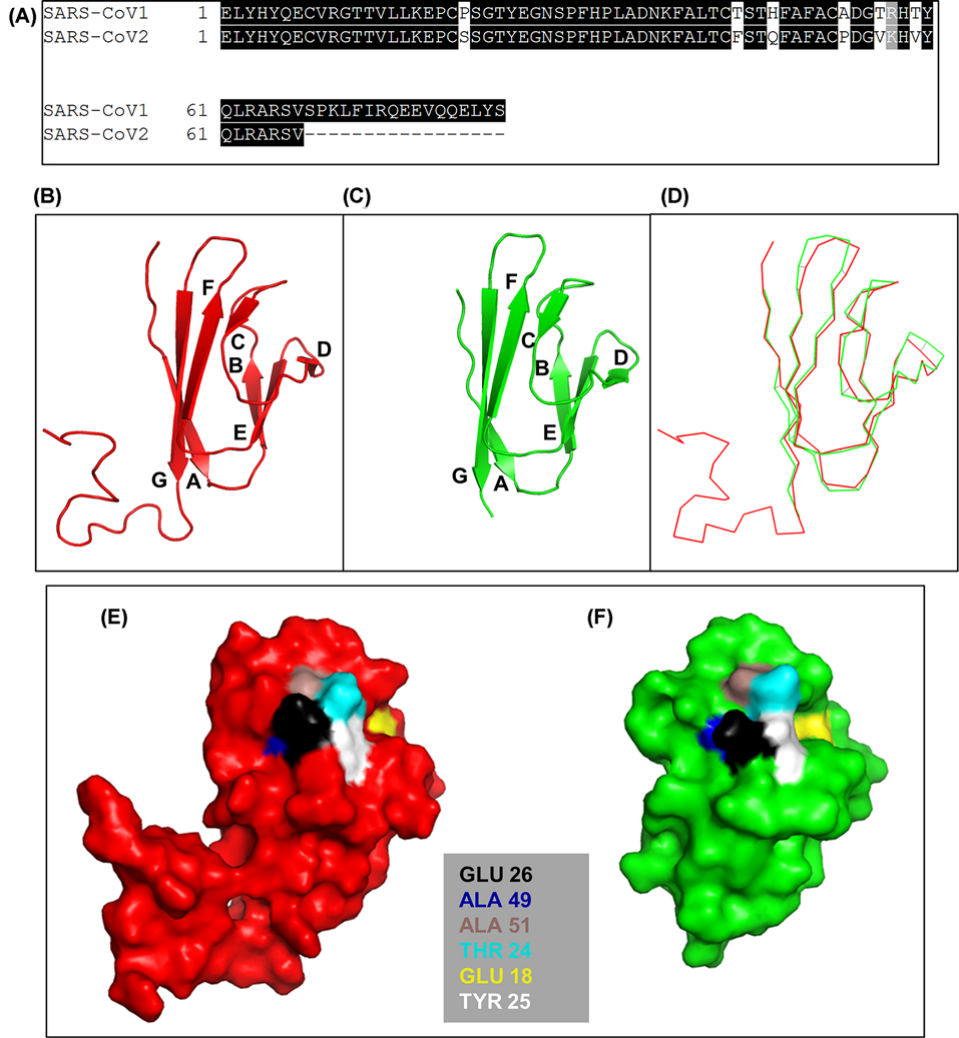
Sequence and structural similarity between the ORF7a proteins of SARS-CoV1 and SARS-CoV2 (**A**) Amino acid sequence pairwise alignment. (**B–D**) 3D structural comparison of ORF7a proteins in (B) SARS-CoV1 (red) and (C) SARS-CoV2 (green) shown in cartoon format. (D) Merge of (B) and (C) in ribbon format. (**E** and** F**) 3D surface map of LFA-1 binding site (Glu26) in ORF7a of (E) SARS-CoV1 (PDB: 1yo4.1.A) and (F) SARS-CoV2 (PDB: 6w37.1.A). Characteristic hydrophobic domains required for LFA-1 binding are also listed and located. All structural images were produced using PyMOL software.

The ORF7a amino acid sequence of SARS-CoV1 (NP_828857.1) and SARS-CoV2 (YP_009724395.1) were compared and shown to be 85% similar (89% identical shared sequence but with 21% fewer amino acids, Figure 1A). Next, we performed 3D structural alignment of the SARS-CoV2-ORF7a protein (PDB template: 6w37) using PDBeFold (https://www.ebi.ac.uk/msd-srv/ssm/) which showed 89% identity (60/67 residues identical) with SARS-CoV1's ORF7a protein (PDB template: 1yo4) (Figure 1B–D) (Table 1).

**Table 1:** PDBe 3D structural alignment summary of SARS-CoV1-ORF7a and SARS-CoV2-ORF7a

##	Q-score	P-score	Z-score	RMSD	Nalgn	Nsse	Ngaps	Seq-%	Nmd	Nres-Q	Nsse-Q	Nres-T	Nsse-T	Query	Target
1	0.6892	11.19	10.12	0.952	66	6	0	0.8939	0	87	6	66	7	PDB 1yo4:A	PDB 6w37:A

Next, we checked whether the key LFA-1 integrin binding determinants present in SARS-CoV1-ORF7a protein were conserved in SARS-CoV2-ORF7a protein as well. Glu34 of the β-strand C was predicted to be the key residue for the interaction of ICAM-1 for LFA-1 binding (Figure 1B) forming a direct coordination to the Mg^2+^ ion of the MIDAS in the LFA-1 I-domain [4,22]. A characteristic feature of the I-domain binding site in ICAM-1 present in SARS-CoV1-ORF7a is a ring of hydrophobic residues around Glu34 which are Pro36, Tyr66, Met64, and the aliphatic portions of Gln62 and Gln73 as well as the basic residue Lys39 [4,22]. This feature was present in SARS-CoV1-ORF7a protein as Glu26 surrounded by hydrophobic residues Ala49, Ala51, Thr 24, Glu18 and Tyr25 (Figure 1E), as previously reported [4]. We show here that this feature is conserved in SARS-CoV2-ORF7a (Figure 1F) [4].

### Molecular docking modelling of ORF7a protein with LFA-1 and Mac-1

**Figure 2:**
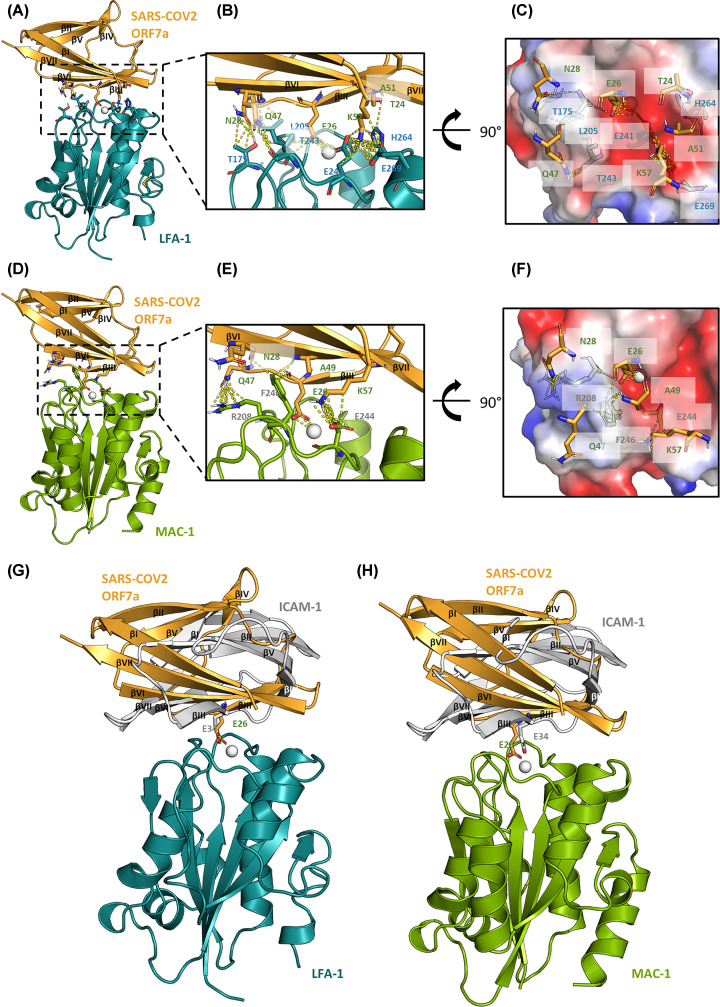
Molecular docking of SARS-CoV2 ORF7a crystal structure with LFA-1 and Mac-1 I domain structures (**A**) Cartoon diagram of the docked complex of the crystal structures of SARS-CoV2 ORF7a (PDB code 6w37) and the LFA-1 I domain (1t0p) with key residues at the binding interface shown as sticks and the Mg^2+^ ion shown as sphere. The β-sheets in SARS-CoV2 ORF7a are numbered N’ to C’. (**B**) Zoomed-in view of the binding interface with the key residues labelled and interactions shown as yellow dashed lines. (**C**) Charged surface representation of the binding interface at a 90° rotated view. (**D**–**F**) Docked complex of SARS-CoV2 ORF7a with the Mac-1 I domain (1ido) in similar representations as in (A, B, C). (**G** and **H**) Superimposition of the crystal structure of the LFA-1:ICAM-1 complex (1mq8) with the docked structures of LFA-1:ORF7a (G) and Mac-1:ORF7a (H) complexes. The key glutamic acids of E26 in ORF7a and E34 in ICAM-1 are shown as sticks.

To investigate the potential binding interfaces of SARS-CoV2 ORF7a with leukocytes, we performed molecular docking experiments with ORF7a and the LFA-1 and Mac-1 I domains. Calculations exploring all orientations resulted in only one orientation with a high score, shown in Figure 2. As predicted, E26 (Glu26) in SARS-CoV2 ORF7a appears to play a critical role in both interactions with LFA-1 and Mac-1, coordinating to the MIDAS Mg^2+^ ion while forming hydrogen bonds with the main-chain of L205 in LFA-1 and R208 in Mac-1. Another charged residue K57 in SARS-CoV2 ORF7a drives electrostatic interactions with E241 and E269 in LFA-1 and E244 in Mac-1. Other main-chain:side-chain hydrogen bonds also contribute to the interaction, including ORF7a N28:LFA-1 T175, ORF7a Q47:LFA-1 T243, ORF7a T24:LFA-1 H264, ORF7a A47:Mac-1 R208, ORF7a N28:Mac-1 F246 and ORF7a A49:Mac-1 F246.

The SARS-CoV2 ORF7a adopts a unique orientation when compared with ICAM-1 (Figure 2G,H). They are both composed of 7 β-sheets, with a glutamic acid residue driving their interactions with the MIDAS site in LFA-1. Consistent with previous predictions [4], ORF7a E26 aligns well with ICAM-1 E34. However, βIII of ORF7a is rotated by 120 degrees in its relative orientation compared to βIII of ICAM-1, whereas ICAM-1 adopts very similar orientations when bound to LFA-1.

The above observations predict that SARS-CoV2-ORF7a protein could act as a binding target for LFA-1 and Mac-1, and thereby propose a likely interaction between SARS-CoV2 and leukocytes, potentially leading to modifications of the immune response.

## Discussion

It is still unclear how SARS-CoV2 modulates the human immune system, causing the severity in disease condition, followed by fatal consequences. Recent clinical reports show that altered lymphocyte and macrophage activity is evident in severe COVID patients [23–28], although the mechanism is largely unclear. Here, we have used in silico molecular docking to predict direct interaction of the SARS-CoV2 viral protein ORF7a with the leukocyte markers LFA-1 and Mac-1. Whether the predicted LFA-1/ORF7a and/or Mac-1/ORF7a interactions modulate SARS-CoV2 behaviour and disease progression requires determination by direct experimental evidence.

Previous work on SARS-CoV1 has confirmed that ORF7a can bind to Jurkat T-cell line expressing LFA-1 [15], and histopathological reports from autopsies have confirmed SARS-CoV1 genome in lymphocytes, macrophages, and monocytes of SARS-infected patients [29]. Consistently, recent histopathological case studies from post-mortem COVID-19 patient tissues have reported the reactivity of SARS-CoV2 spike protein antibody in tracheal submucosa lymphocytes and alveolar macrophages [30], with observations of the splenic and lymph node regions of severe COVID patients have shown significant lymphocytic apoptosis, and SARS-CoV2 nucleoprotein reactivity along with IL-6 upregulation in ACE2 positive macrophages [31]. A recent study has confirmed SARS-CoV2 can directly infect and replicate in CD4 T helper cells via viral infection tests of isolated healthy human peripheral blood samples, and by confirming presence of SARS-CoV2 RNA in COVID patients [32]. However, these studies have not confirmed the expression of LFA-1 or Mac-1 in the SARS-CoV2-infected cells.

Recent studies have also reported significant reduction in circulating LFA-1 expressing T lymphocytes [33], a significant increase in Mac-1 expression in monocytes [34,35], and a significant decrease of Mac-1 expression in granulocytes [36] in symptomatic COVID patients, but no confirmation on whether these cells were infected with SARS-CoV2. Both these observations could be consistent with our predictions and require further *in vitro* receptor binding assays coupled with histopathological studies from infected cells and tissues to robustly confirm the infectibility of SARS-CoV2 in LFA-1 and Mac-1 expressing cells, and the role of ORF7a in modulating LFA-1 and Mac-1 expressing cells. It should be noted that LFA-1 and Mac-1 expression is shared by multiple types of leukocytes [37], and therefore it would be interesting to determine if ORF7a protein modulates the function of LFA-1/Mac-1 expressing leukocytes differentially based on cellular heterogeneity. Also, recent studies have reported three mutations in the SARS-CoV2 genome in infected patients where large deletions in the ORF7a gene have been recorded, although the LFA-1 binding site residue Glu26 and the following sequence are conserved in all mutants, suggesting an indispensable functional site [38,39].

Based on the physical subcellular localisation of the SARS-CoV2-ORF7a protein and published reports of SARS-CoV1 ORF7a behaviour [5,40–46], there are two possible ways by which it could influence disease progression in COVID-19 patients. Firstly, ORF7a protein present on the viral membrane could interact with the spike protein or directly bind to LFA-1/Mac-1 present on the cell membrane of leukocytes, thereby modulating cell targeting. Western blot analysis of SARS-CoV1 particles using sucrose density ultracentrifugation or viral capture assay detected the presence of ORF7a protein, confirming its presence as a viral structural protein [46]. Secondly, intracellular accumulation of ORF7a protein could restrict translocation of LFA-1/Mac1 integrins to the cell membrane and reduce LFA-1/Mac-1 dependent cell signalling, an important cascade in leukocyte function. One study has confirmed the intracellular localisation of ORF7a protein in the endoplasmic reticulum and Golgi compartments of ORF7a cDNA transfected and SARS-CoV1 infected cells [5]. Other studies have reported that *in vitro* expression of ORF7a results in apoptosis through a caspase-dependent pathway, inhibition of cellular protein synthesis, blockage of cell cycle progression at G0/G1 phase, activation of NF-kB, increased IL-8 promoter activity, and activation of p38 MAP kinase [41–45]. These varied results all suggest a role for ORF7a in altering the host cellular environment and future experimental plans should include assessment of ORF7a activity and binding mechanisms to leukocytes as a structural, intracellular and soluble protein, analysis of resulting cell viability and cytokine release, and testing of FDA-approved LFA-1/Mac-1/ICAM-1 inhibitors for their effect on SARS-CoV2 infection in both ACE2 and LFA-1/Mac-1 expressing cells *in vitro* [40,47].

## Data Availability

There are no mandated datasets provided within the paper.

## Abbreviations

ACE2: angiotensin 2
AIR: ambiguous interaction restraint
ICAM-1: intercellular adhesion molecule-1
LFA-1: lymphocyte function-associated antigen-1
MIDAS: metal ion-dependent adhesion site
ORF7a: open reading frame 7a
SARS-CoV2: severe acute respiratory syndrome coronavirus 2
